# Operations research to add postpartum family planning to maternal and neonatal health to improve birth spacing in Sylhet District, Bangladesh

**DOI:** 10.9745/GHSP-D-13-00002

**Published:** 2013-08-14

**Authors:** Salahuddin Ahmed, Maureen Norton, Emma Williams, Saifuddin Ahmed, Rasheduzzaman Shah, Nazma Begum, Jaime Mungia, Amnesty Lefevre, Ahmed Al-Kabir, Peter J Winch, Catharine McKaig, Abdullah H Baqui

**Affiliations:** a Johns Hopkins Bloomberg School of Public Health, Baltimore, MD, USA; b Jhpiego, Baltimore, MD, USA; c U.S. Agency for International Development, Washington, DC, USA; d Research, Training and Management (RTM) International, Dhaka, Bangladesh

## Abstract

**Background:**

Short birth intervals are associated with increased risk of adverse maternal and neonatal health (MNH) outcomes. Improving postpartum contraceptive use is an important programmatic strategy to improve the health and well-being of women, newborns, and children. This article documents the intervention package and evaluation design of a study conducted in a rural district of Bangladesh to evaluate the effects of an integrated, community-based MNH and postpartum family planning program on contraceptive use and birth-interval lengths.

**Intervention:**

The study integrated family planning counseling within 5 community health worker (CHW)-household visits to pregnant and postpartum women, while a community mobilizer (CM) led community meetings on the importance of postpartum family planning and pregnancy spacing for maternal and child health. The CM and the CHWs emphasized 3 messages: (1) Use of the Lactational Amenorrhea Method (LAM) during the first 6 months postpartum and transition to another modern contraceptive method; (2) Exclusive, rather than fully or nearly fully, breastfeeding to support LAM effectiveness and good infant breastfeeding practices; (3) Use of a modern contraceptive method after a live birth for at least 24 months before attempting another pregnancy (a birth-to-birth interval of about 3 years) to support improved infant health and nutrition. CHWs provided only family planning counseling in the original study design, but we later added community-based distribution of methods, and referrals for clinical methods, to meet women's demand.

**Methods:**

Using a quasi-experimental design, and relying primarily on pre/post-household surveys, we selected pregnant women from 4 unions to receive the intervention (n = 2,280) and pregnant women from 4 other unions (n = 2,290) to serve as the comparison group. Enrollment occurred between 2007 and 2009, and data collection ended in January 2013.

**Preliminary Results:**

Formative research showed that women and their family members generally did not perceive birth spacing as a priority, and most recently delivered women were not using contraception. At baseline, women in the intervention and comparison groups were similar in terms of age, husband's education, religion, and parity. CHWs visited over 90% of women in both intervention and comparison groups during pregnancy and the first 3 months postpartum.

**Discussion:**

This article provides helpful intervention-design details for program managers intending to add postpartum family planning services to community-based MNH programs. Outcomes of the intervention will be reported in a future paper. Preliminary findings indicate that the package of 5 CHW visits was feasible and did not compromise worker performance. Adding doorstep delivery of contraceptives to the intervention package may enhance impact.

## Abstract (precis)

This quasi-experimental study integrated family planning, including the Lactational Amenorrhea Method, into community-based maternal and newborn health care and encouraged transition to other modern methods after 6 months to increase birth-to-pregnancy intervals. Community-based distribution of pills, condoms, and injectables, and referral for clinical methods, was added to meet women's demand.

**BOX. Communication Messages Specific to Birth Spacing**

**Recommendations on Healthy Timing and Spacing of Pregnancy**

- **24 months between pregnancies**
  After a live birth, wait at least 24 months before attempting the next pregnancy to reduce the risk of adverse maternal, perinatal, and infant outcomes.
- **6 months following miscarriage/induced abortion**
  After a miscarriage or induced abortion, wait at least 6 months before attempting the next pregnancy to reduce risks of adverse maternal and perinatal outcomes.

**Health Outcomes Related to Short Birth Intervals**

- **Less than 24 months from the last live birth to the next pregnancy**
  1. Newborns can be born too soon, too small, or with a low birth weight.
  2. Infants and children may not grow well and are more likely to die before the age of 5.
- **Less than 6 months from the last live birth to the next pregnancy**
  1. Mothers may die in childbirth.
  2. Newborns can be born too soon, too small, or with a low birth weight.
  3. Infants and children may not grow well and are more likely to die before the age of 5.
- **Less than 6 months after a miscarriage or abortion to the next pregnancy**
  1. Mothers are at higher risk of developing anemia or premature rupture of membranes.
  2. Newborns can be born too soon, too small, or with a low birth weight.

**Benefits of Healthy Timing and Spacing of Pregnancies**

- **For newborns, infants, and children under 5**
  1. Reduced risk of preterm births, low birth weight, small for gestational age, and, in some populations, stunting or underweight conditions.
  2. Reduced risk of death for newborns, infants, and children under 5.
  3. Increased chance that children will experience the health benefits of breastfeeding for a full 2 years.
- **For mothers**
  1. More time to prepare physically, emotionally, and financially for the next pregnancy (if desired).
  2. For young mothers, reduced risk of pregnancy-induced high blood pressure and associated complications, obstructed or prolonged labor, iron deficiency anemia, and maternal death.
  3. More time to focus on infant, partner, and other children.
  4. Reduced risk of pregnancy complications, such as preeclampsia.
  5. May increase duration of breastfeeding, which is linked with reduced risk of breast and ovarian cancer.
- **For men**
  1. Helps men safeguard the health and well-being of their partners and children.
  2. Allows men time to plan financially and emotionally for their next child (if desired).
  3. Contributes to a man’s sense of satisfaction from supporting his partner in making healthy decisions regarding raising a healthy family.
- **For communities**
  1. Reduces deaths and illnesses among mothers, newborns, infants, and children.
  2. Helps to reduce poverty and improve the quality of life among community residents.

## BACKGROUND

During the last several decades, global family planning programs have contributed to an increase in contraceptive prevalence and to a decrease in total fertility rates (TFRs) worldwide.^1^ In Bangladesh, family planning programs have contributed to a decline in the TFR between 1975 and 2007, from 6.3 to 2.7 children per woman.^2^-^3^ The country was often cited as a family planning success story in the 1990s for improving contraceptive prevalence, from 3% in 1971 to 40% by 1991.^4^-^5^ Currently, 52% of married women in Bangladesh use any modern contraception.^6^

Promoting contraceptive use immediately after birth is considered an important programmatic strategy, but it remains a major challenge. The Population Council first launched a global postpartum family planning program in 1966,^7^ implemented largely through hospitals because most contraceptive methods available at that time were clinic-based (notably, IUDs and sterilization). The vast majority of women in developing countries, however, were delivering at home. Thus, the program failed to reach the critical mass required for its success, and it officially ended in 1974. More recently, the World Health Organization (WHO), the U.S. Agency for International Development (USAID), and other partners have issued a joint statement for collective action emphasizing that programs should “prioritize reaching postpartum women, the group of women with the greatest unmet need for family planning.”^8^

Many family planning programs historically have focused on improving access to family planning and on changing people's attitudes about ideal family size and reducing the TFR, rather than on promoting longer birth intervals. In low-income countries, approximately 57% of second and higher-order births occur at intervals shorter than 3 years, and, in some countries, these conditions have not changed in 20 years.^9^

Consistent observational data from many settings suggest that birth-to-pregnancy intervals shorter than 24 months (equivalent to birth-to-birth intervals of 33 months, or about 3 years) are associated with increased risk of poor maternal, perinatal, and neonatal health outcomes, including increased risk of stillbirth, prematurity, low birth weight, and neonatal mortality.^10^-^14^ Studies in Bangladesh specifically have found that pregnancy intervals shorter than 36 months are associated with a 37% increased risk of late neonatal mortality and 23% increased risk of child mortality, relative to intervals of 36–59 months.^15^ In addition, birth-to-pregnancy intervals of 15–75 months are associated with a lower likelihood of fetal loss than intervals greater than 75 months or shorter than 15 months.^16^ With respect to women's health, studies in Bangladesh have found that, after adjustment, birth-to-pregnancy intervals shorter than 6 months are associated with increased risk of pre-eclampsia, as well as a 7.5-fold increased risk of induced abortion and a 3.3-fold increased risk of miscarriage, compared with intervals of 27–50 months.^16^-^17^

Short birth intervals are associated with poor maternal and newborn health outcomes.

Policymakers have called for better integration of family planning services with maternal and child health programs,^18^-^19^ but notable challenges exist in attaining this goal.^20^ For instance, in Bangladesh, the Ministry of Health and Family Welfare has separate divisions for “Health Services” and “Family Planning” with separate directorates and cadres of workers, complicating efforts to integrate services.^21^

A 2012 Cochrane review did not find any integrated family planning and maternal, newborn, and child health program in a developing country that had operationalized the growing evidence on the role of family planning in improving child and maternal health (by strengthening counseling on the benefits of longer birth-to-pregnancy intervals, for example).^22^ Although the review noted that improvements in mortality and morbidity outcomes did occur in some integrated programs, the studies did not document clearly the implementation and program design details.

In this article, we describe the intervention package and evaluation design of an operations research study that is nearing completion, called the Healthy Fertility Study (HFS). The study integrated postpartum family planning education and services within an existing community-based maternal and neonatal health (MNH) program, called “Projahnmo” (Project for Advancing the Health of Newborns and Mothers), to improve contraceptive knowledge and practices and birth spacing, focusing specifically on using the Lactational Amenorrhea Method (LAM) during the first 6 months postpartum and transitioning to another modern method afterward. Projahnmo uses female community health workers (CHWs) to deliver MNH services through home visits, complemented by community mobilization activities. The Projahnmo program reduced neonatal mortality by 34% in its first 30 months of implementation in Sylhet district, located in the Sylhet division of rural, northeastern Bangladesh.^23^ HFS targeted family planning services to pregnant and postpartum women because studies have found that two-thirds of women who are within 1 year of their last birth have an unmet need for family planning.^24^

Many postpartum women have an unmet need for family planning.

## STUDY AREA

We chose to conduct the study in Sylhet division because it had the highest neonatal, infant, and under-5 child mortality rates among the 7 divisions of Bangladesh.^3^ In addition, at the time of the design, the division ranked poorly on indicators related to contraceptive use and fertility reduction,^3^ including:

- TFR of 3.7 compared with 2.7 for Bangladesh overall
- Unmet need for contraception of 26% compared with 17% for Bangladesh overall
- 57% of second and higher-order births occurred less than 36 months after the preceding birth compared with 37% for Bangladesh overall

Nonetheless, the presence of government-run health and family planning facilities and exposure to family planning messages in the media in Sylhet division are comparable with other parts of Bangladesh. However, women's access to clinical family planning services in Sylhet district is severely constrained. One-quarter of the public-sector positions slated for provision of clinical family planning services are unfilled (personal communication with Mr. Kutub Uddin, Divisional Director of Family Planning, Sylhet Division, Ministry of Health and Family Welfare, Aug 2012). When clinical family planning services are available, they are offered only at specific times during the week. Nongovernmental and private-sector providers also largely lack clinical skills in family planning. Equally important, in Sylhet, cultural norms restrict women from leaving their homes without a chaperone.

## INTERVENTION DESCRIPTION

### Formative Research to Design Service Delivery Strategies

We conducted formative research between January 2006 and June 2006 to inform the intervention design for postpartum family planning service delivery. Formative research activities included:

- 80 unstructured, in-depth interviews with household members (mothers of children ages 6–23 months with a preceding birth interval longer than 36 months or shorter than 18 months, husbands of these mothers, and elder women living in the home, usually mothers-in-law) to elicit information on birth spacing, timing of first birth, contraceptive practices, and other issues related to healthy fertility
- 40 semi-structured interviews with mothers who practice and who do not practice birth spacing and their mothers-in-law, using free-listing exercises in which participants listed the benefits of birth spacing and risks associated with short birth intervals
- 6 focus groups with mapping exercises among recently delivered women (3) and older women (3) to explore community perceptions about birth spacing, as well as to compile a list of health care providers who offered family planning services
- A household survey with 1,612 systematically sampled, recently delivered women to gather data on household sociodemographic factors, contraceptive knowledge and practices, and access to and use of health services
- 31 semi-structured interviews with health care providers and 4 focus group discussions with community opinion leaders, including religious leaders, members of Union Councils (local-level administrative bodies), and village government leaders, to obtain their opinions on healthy timing and spacing of pregnancies

Interviewers transcribed and coded the interview and focus group discussions, and the research team reviewed the transcripts and codes to identify themes and relevant findings. When religious leaders understood that family planning was being integrated to support women's and children's health, they stated that they had no objection to this activity.

#### Perceptions of Birth Spacing Among Community Members

All 40 of the mothers and mothers-in-law that participated in the free-listing exercises were able to list at least 4 risks to either the mother or child associated with closely spaced births. Similarly, 38 of the 40 participants were able to list at least 3 benefits associated with a healthy birth-spacing interval, including physical, emotional (less mental stress), and economic benefits.

Nearly 78% of recently delivered women said that they desired a birth-spacing interval of 3 years or longer. During in-depth interviews, some men stated an economic interest in spacing births, and some were also aware of the physical toll that closely spaced births would have on the health of the mother.

Respondents generally did not perceive birth spacing as a high priority relative to other family needs. Mothers-in-law considered birth spacing of secondary importance to the provision of grandchildren, which they saw as a necessary and critical obligation of their daughters-in-law. Mothers themselves also said that birth spacing was not a very serious issue for them. Having many children within short intervals is common and perceived as normal in their communities; as a result, the women had not questioned the practice previously. Many of the husbands interviewed considered birth spacing as more important to their wives than to them.

Community members could identify benefits of longer birth intervals, but birth spacing generally was not a high priority for them.

#### Contraceptive Use Among Recently Delivered Women

Despite recognition of the risks of closely spaced births and the benefits of longer intervals, nearly 75% of the recently delivered women (who were not sterilized) were not using any contraceptive method to prevent pregnancy.

#### Knowledge of LAM Among Providers

Among health care providers, family planning service delivery reportedly focused more on limiting family size than on advocating birth spacing. Knowledge of LAM, including its objectives and criteria, was limited. (Three criteria for LAM must be met to provide effective protection against pregnancy: the mother's menstrual periods have not returned; the baby is fully or nearly fully breastfed and is breastfed often, day and night; and the baby is less than 6 months old.^25^)

### Service Delivery Strategies

Based on the findings that most women recognized the risks of short birth intervals and benefits of longer intervals, and that most wanted birth-spacing intervals of 3 years or longer, we developed a strategy that:

- More clearly specifies the healthy behaviors that women and couples need to practice to achieve these goals
- Reinforces risks and benefits
- Provides integrated family planning and MNH services, drawing upon existing cadres of community health workers (CHWs). Each CHW serves a population of about 4,000, or 4 villages.

The CHWs are young women from the local community with grade 10 education. All CHWs in both intervention and comparison areas received 21 days of basic MNH training, including skills development for behavior change communication, clinical assessment of neonates, and hands-on clinical training under supervision in a tertiary care hospital and in households. CHWs in the intervention area received an additional 3 days of training about healthy timing and spacing of pregnancy and postpartum family planning, including LAM, and 4.5 days of training about contraceptive methods and logistics management, according to protocols from the government of Bangladesh.

#### Timing of CHW Visits

In the existing Projahnmo MNH program, CHWs visited pregnant and postpartum women 6 times during the antenatal and postpartum period. They also made 1 visit every 2 months within their catchment area to identify pregnant women.

In the intervention area, the HFS integrated postpartum family planning counseling within 3 of the 6 existing MNH visits: at 30–32 weeks of pregnancy, 6 days postpartum, and once between days 29–35 days postpartum. The study also added 2 more visits, at 2–3 months and 4–5 months postpartum (Table 1), to educate families about postpartum family planning and provide services as needed.

**Table 1.** Postpartum Family Planning Communication Messages, by Timing of CHW Visits

	CHW Visits Within Existing MNH Visits			New CHW Visits	
Communication Messages	During Pregnancy	On Day 6 Postpartum	Between Days 29–35 Postpartum	Between Months 2–3 Postpartum	Between Months 4–5 Postpartum
Benefits of longer birth intervals, risks of shorter birth intervals	✓	✓	✓	✓	✓
Essential newborn care, including exclusive breastfeeding	✓	✓	✓		
LAM, promotion of 6 months of exclusive breastfeeding	✓	✓	✓	✓	✓
Timing of return to fertility, signs indicating return to fertility			✓	✓	✓
Transition from LAM to another modern contraceptive method			✓	✓	✓
Discussion of contraceptive methods, potential side effects, strategies to minimize side effects			✓	✓	✓
Referral to health facility for contraceptive methods, if needed			✓	✓	✓

Abbreviations: CHW, community health worker; LAM, Lactational Amenorrhea Method; MNH, maternal and neonatal health.

#### Family Planning Interventions to Support Improved Newborn and Child Survival

We designed 3 family planning interventions specifically to support improvements in newborn and child health. The purpose was to ensure that adding family planning activities to the existing MNH services did not undermine program achievements to date in newborn health outcomes.

1. **Use of LAM during the first 6 months postpartum to prevent high-risk pregnancies.** LAM can be considered a maternal and child survival intervention because its use prevents high-risk pregnancy during the critical 6-month period after a birth. A recent meta-analysis showed that infants conceived during the first 6 months after a live birth are at elevated risk of multiple adverse outcomes.^14^ Studies in multiple settings have shown LAM to be 98% effective, including with limited client contact.^26^-^28^ In addition, a multicenter study found that two-thirds of LAM users eventually accept another modern contraceptive method.^28^ In this largely rural region of Sylhet, where women's access to health facilities and to modern contraception is highly constrained, the HFS team decided to promote LAM use as a “gateway” method of family planning to address unmet need and to prevent high-risk pregnancies during the critical 6-month period after a birth. In the HFS intervention areas, CHWs always referred to LAM as “LAM and Transition,” emphasizing the importance of transitioning at 6 months postpartum to another modern method.
2. **Use of LAM to increase the duration of exclusive breastfeeding and improve breastfeeding practices.** LAM use is associated with improved exclusive breastfeeding practices. At the time of the HFS design, the median duration of exclusive breastfeeding in Sylhet division was less than 1 month (0.7).^29^ Although LAM requires women to be *fully or nearly fully* breastfeeding, the HFS team decided to communicate the breastfeeding criterion of LAM as *exclusive* breastfeeding, to prevent the possibility of undermining exclusive breastfeeding practices promoted by the ongoing MNH program. The team hoped to replicate results achieved in Jordan, where one study found that use of LAM promoted good exclusive breastfeeding practices.^30^ The team also hoped that promoting use of LAM would increase the duration of exclusive breasfeeding.
3. **Use of other modern methods to achieve a 24-month birth-to-pregnancy interval, which is associated with reduced risk of under-5 mortality and improved maternal and child nutrition status.** Nearly half of all contraceptive users in Bangladesh stop using their method within 12 months of starting.^29^ A global study found that high discontinuation rates were linked with low motivation to avoid pregnancy.^31^ The HFS team explored whether conveying information to clients about the health risks to their infants posed by rapid, repeat pregnancies would be sufficient to motivate women to continue with a method for at least 24 months, or longer, after a live birth. We based communication messages on recommendations from a 2005 World Health Organization (WHO) Technical Consultation on Birth Spacing. The communication messages clearly specified the healthy behavior—that is, after a live birth, to use a modern contraceptive method for at least 24 months before attempting a pregnancy.^32^ Risk information was conveyed in simple and easy-to-understand language; for example, “if the pregnancies are too close together, the baby could be born too soon or be too small.”^32^ We tailored messages to our target audiences of women, men, and communities, and we were as specific as possible to improve the potential for positive behavior change (Box).^33^

LAM is often referred to as a “gateway” method of family planning, because many LAM users eventually accept another modern method.

#### Development of Communication Materials

After conducting a desk review of existing information, education, and communication (IEC) materials in Bangladesh from NGOs and the government, we adapted materials on postpartum care from the Bangladesh Rural Health Service Delivery Program and developed, field tested, and refined into the local language 3 new leaflets on exclusive breastfeeding and birth spacing, LAM, and return to fertility after delivery (see supplementary material):

- **Postpartum care:** A pictorial description of postpartum services and potential postpartum complications, and the importance of visiting a health center at the sign of any complication. The leaflet also emphasizes visiting a health center for a postpartum physical check-up, for immunization of the baby, and to choose a contraceptive method.
- **Exclusive breastfeeding and birth spacing:** Describes exclusive breastfeeding, the benefits of birth-to-pregnancy spacing of at least 24 months, the citation from the Quran about breastfeeding, and visiting a health facility for consultations.
- **LAM**: A pictorial listing of LAM criteria and when to transition, and the importance of timely transition, to another modern method when LAM is no longer effective.
- **Return to fertility:** Describes the story of a woman named Asma; the story discusses the short time period before fertility returns after delivery, and variations in return to fertility from woman to woman, including messages describing the risk of becoming pregnant prior to the return of menses and as soon as 1 month postpartum if the baby is not breastfed, as well as the benefits of birth-to-pregnancy spacing of at least 24 months. The story was received well by women in the study, many of whom could relate to it.

#### Community-Based Distribution of Methods Added to Study Design

In the original study design, CHWs provided family planning counseling based on women's fertility intentions—whether and when women would like to have another child, or whether they would prefer to end childbearing—but they did not distribute contraceptive methods. There was demand among women for contraceptives but they had difficulty accessing them from health facilities.

After receiving approval from the JHU Institutional Review Board in July 2009 to amend the study protocol, the HFS team began training CHWs to distribute combined hormonal contraceptive pills and condoms, in accordance with Bangladeshi government protocols for in-home provision of contraceptives by community-based workers. That is, CHWs provided pills only after they completed verbal screening for medical eligibility to rule out risk factors that would preclude the woman from using the method. (The progestin-only pill is the preferred oral contraceptive during early breastfeeding; however, it was unavailable in Bangladesh until approximately January 2011 when it was introduced through private-sector health services.) CHWs distributed pills and condoms during their routine pregnancy surveillance visits every 2 months.

Community-based distribution of contraceptives was added to the study design to meet women's demand.

Beginning in March 2011, CHWs also started providing progestin-only injectable contraceptives to women enrolled in the HFS. Women must receive the injection every 3 months, but they have a window period and can receive the injection up to 2 weeks early or up to 4 weeks late (according to government of Bangladesh protocol and international guidance^34^-^35^). CHWs maintained a list of all injectables users in their planning book, in which they noted the injection receipt date and the next scheduled date. During their regular pregnancy surveillance visits in the community or during community visits for antenatal or postpartum monitoring, CHWs provided injections to injectables users if the dates matched. CHWs also made *active* referrals for clinical contraceptive methods—that is, CHWs accompanied women desiring clinical methods to the facility.

#### Community Mobilization and Education

In addition to the one-on-one counseling provided by CHWs, male and female community mobilizers organized monthly meetings at the cluster level with pregnant and postpartum women, their husbands, mothers-in-law, and fathers-in-law to discuss the importance of birth-spacing practices and postpartum family planning, including LAM and return to fertility after delivery. At the meetings, the mobilizers recognized women who practiced LAM successfully, some of whom were designated as “LAM Ambassadors.” LAM Ambassadors provided information and support to pregnant and postpartum women in their communities about the value of LAM. Their healthy and well-spaced babies appeared to be an effective advertisement for the healthy family planning behaviors advocated by HFS.

Community mobilizers also conducted advocacy meetings with local leaders, including religious leaders, teachers, businessmen, government, and NGO staff, to sensitize the community about study activities.

**Figure f02:**
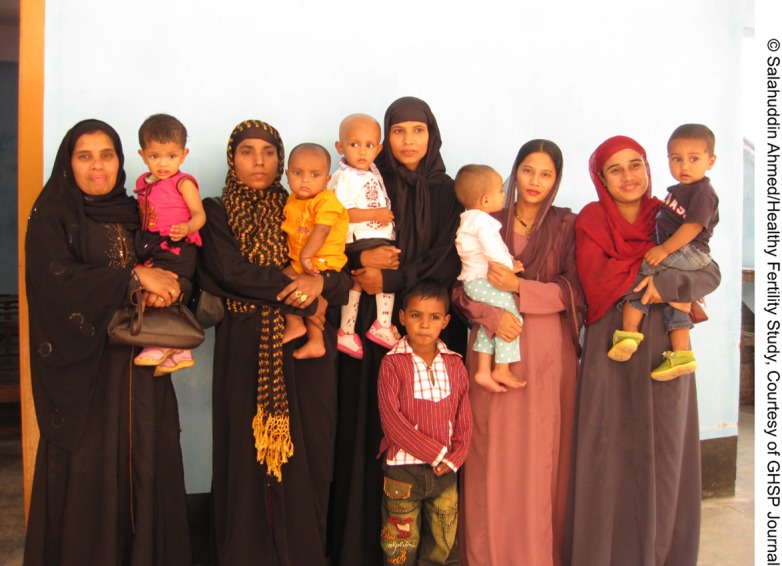
The Healthy Fertility Study used successful LAM users, designated as “LAM Ambassadors,” to promote LAM to other pregnant and postpartum women in their communities.

## METHODS

### Study Design

In December 2007, enrollment of pregnant women began in 4 unions that had ongoing maternal and neonatal health programs. (A union is the lowest administrative unit with, on average, a population of 25,000 people and a primary health care facility.) We intended to enroll women over a 7-month period (through June 30, 2008), but we received additional funding that allowed us to increase the study area to 8 unions, thus expanding the enrollment period through July 14, 2009 (Figure).

**Figure:**
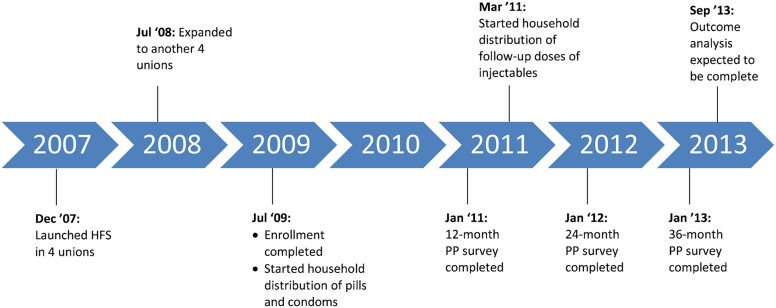
Timeline: Implementation of Healthy Fertility Study Abbreviations: HFS, Healthy Fertility Study; PP, postpartum.

Using a quasi-experimental design, and relying primarily on pre/post-household surveys among study participants, we purposefully selected 4 unions to receive the intervention and 4 other unions to serve as the comparison group, receiving standard maternal and newborn care promotion only.

The study unions were purposively selected to:

1. Minimize contamination between intervention and comparison groups and with other ongoing nongovernmental organization (NGO) programs
2. Exclude unions having an *Upazila* (sub-district) Health Complex (hospital facility with inpatient and outpatient services), as this would represent a large difference in availability of health services
3. Include unions with functional government facilities and workers designated for providing family planning services

The study was approved by the Johns Hopkins University (JHU) Institutional Review Board and the Bangladesh national ethics committee of Bangladesh Medical Research Council and is registered as a Clinical Trial (Identifier: NCT01702402).

**Figure f03:**
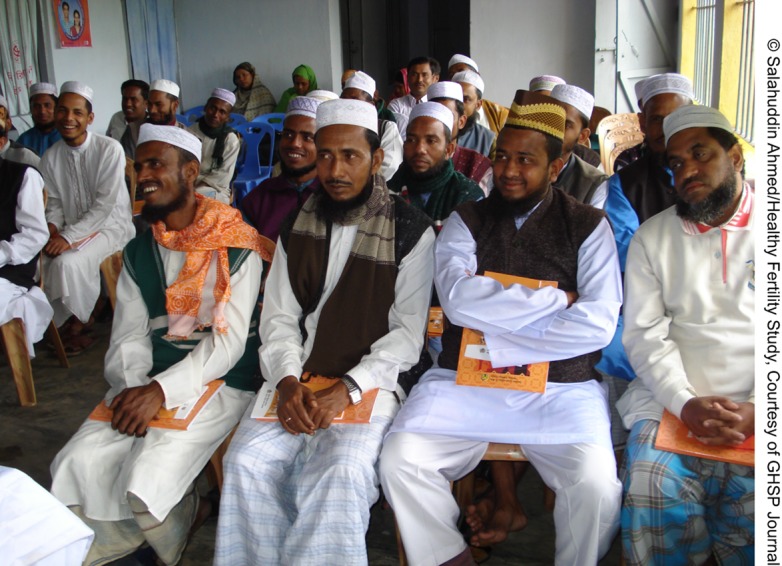
During the Healthy Fertility Study, community mobilizers met with religious leaders during advocacy meetings.

### Sample Size and Outcomes of Interest

Following recommendations from the 2005 WHO Technical Consultation on Birth Spacing,^32^ this intervention promotes knowledge and use of contraceptive methods to increase birth-to-pregnancy intervals to at least 24 months (equivalent to a birth-to-birth interval of 33 months, or almost 3 years) to improve newborn, infant, child, and maternal health outcomes.

Although birth-to-birth intervals can be measured more accurately than birth-to-pregnancy intervals, from a programmatic perspective, it is easier to communicate messages about birth-to-pregnancy intervals, because women and their partners think in terms of the time from the birth of one child to the conception of the next child. Thus, the household survey questionnaires refer to birth spacing in terms of birth-to-pregnancy intervals. However, birth-to-birth interval is the study's primary outcome measure.

To calculate sample size, we hypothesized that the proportion of women having another birth within 33 months of the last birth will be 12% in the intervention area and 16% in the comparison area—a 25% difference. To measure a 25% decrease in the proportion of women with a birth interval shorter than 33 months with 80% power and 5% significance level required a sample size of 1,181 pregnant or postpartum women per study arm. Taking into account a design effect of 1.5, we estimated we would need to increase the sample size to 1,772 women per study arm. We assumed a 20% loss to follow-up (10% per year), which further increased the sample size to 2,215 per arm (4,430 total). This sample size is sufficient to measure hypothesized changes in contraceptive knowledge and use and birth-to-birth intervals of 33 months. The total number of women enrolled was 4,570, slightly higher than anticipated as a result of ongoing field operations in several locations simultaneously (n = 2,280 in intervention group, n = 2,290 in control group). The final study cohort was comprised of 4,504 women (2,247 in intervention and 2,257 in control group) for whom data on pregnancy outcomes were available.

**Figure f04:**
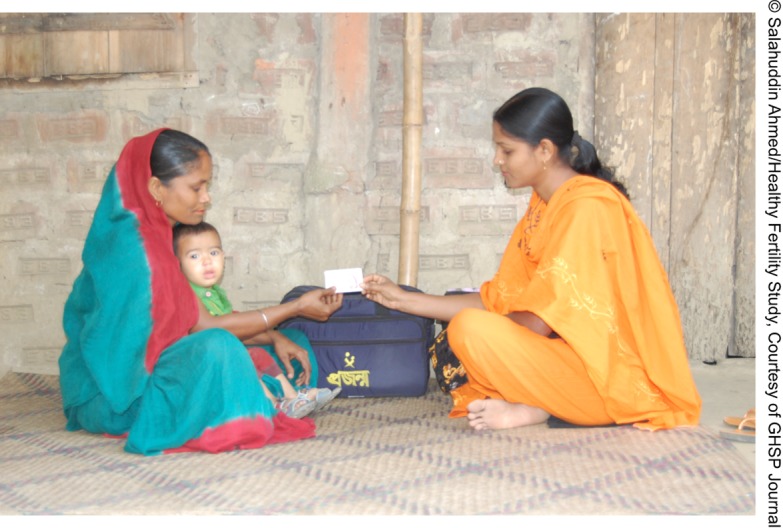
Community health workers provided doorstep delivery of oral contraceptive pills and other modern methods to women in their communities.

### Data Collection

A team of data collectors not involved in implementing the intervention conducted 8 data collection visits for each study participant:

- 1 visit during the antenatal period completed before weeks 30–32 of pregnancy (considered the baseline)
- 7 follow-up visits during the postpartum period (at 3, 6, 12, 18, 24, 30, and 36 months postpartum)

In addition to the independent data collectors, CHWs also collected data used to evaluate the intervention, including date of birth of newborns, birth weight, and neonatal outcomes. A separate team of data collectors conducted verbal autopsies of all reported infant deaths occurring in both study arms.

## PRELIMINARY RESULTS

### Baseline Findings on Enrolled Participants

Women in the intervention and comparison groups were similar in terms of age, husband's education, religion, and parity (Table 2). Women's education was slightly higher in the intervention group than in the comparison group. Levels of ever-use of contraception were slightly lower in the intervention group (18%) than in the comparison group (21%), and fertility desires were slightly higher (wants more children) (60% versus 56%, respectively). Women in the intervention group were comparatively poorer than women in the comparison group, but the difference was not statistically different (*P* = .10).

**Table 2.** Baseline Characteristics of Enrolled Study Participants (N = 4,570)

	Study Group		
Characteristic	Intervention (n = 2,280)	Comparison (n = 2,290)	*P* value
Age, mean (95% CI), y	26.5 (25.4–27.6)	26.6 (26.0–27.3)	.86
Years of schooling, mean (95% CI)			
Women	4.5 (4.1–4.8)	4.1 (3.6–4.5)	.11
Husbands	4.0 (3.4–4.6)	4.0 (3.2–4.7)	.88
Religion			
Muslim	94.5%	91.4%	.40
Hindu/other	5.5%	8.6%	
Parity, mean (95% CI)	2.2 (2.0–2.3)	2.2 (1.9–2.3)	.74
Economic Status, %			
Poorest	18.6	22.6	.10
Poor	16.8	22.8	
Middle	19.5	20.2	
Rich	22.6	17.2	
Richest	22.5	17.3	
Ever used contraception, %	18.0	21.1	.51
Fertility desires, %			
Wants more children	59.7	55.7	.36
Wants no more	26.0	32.4	
Undecided/up to God	14.3	11.9	

Abbreviations: CI, confidence interval.

*P* values are adjusted for clustering effects. *P* values ≤ .05 were considered statistically significant.

### CHW Visits

There was no difference in coverage of women by CHWs in the intervention versus control groups (Table 3). During pregnancy, CHWs visited more than 99% of women in both study groups. During the first 3 months postpartum, CHWs visited about 96% of women in the intervention group and 93% of women in the control group. However, within the first week of delivery, CHWs visited comparatively fewer women in the intervention group than in the control group (89.8% versus 96.4%, respectively; *P*<.05). Still, the mean number of CHW visits was higher in the intervention than in the control group (4.2 versus 3.5, respectively; *P*<.01). This was not unexpected since CHWs provided contraceptive methods to women in the intervention group, which probably required additional CHW visits for resupply of methods.

**Table 3.** Coverage Rates of Community Health Worker (CHW) Visits

	Study Group		
Timing of CHW Visit	Intervention (n = 2,183)	Comparison (n = 2,216)	*P* value
During pregnancy	99.4%	99.6%	.42
Within 3 months postpartum	95.6%	93.0%	.21
Within first week postpartum	89.8%	96.4%	.007
Mean number of visits	4.2	3.5	.001

*P* values ≤ .05 were considered statistically significant.

During pregnancy, denominator is all women with complete information.

During the postpartum period, denominator is all women with a surviving infant at 3 months postpartum.

## DISCUSSION

This article describes the design of an operations research study that integrates postpartum family planning education and services within an existing community-based maternal and newborn care program in a low-resource setting.

The HFS adds to the literature on postpartum family planning. In developed countries, promotion of postpartum family planning is a standard component of obstetric care.^36^ Postpartum family planning is also included in guidelines for health care workers in developing countries, but in practice, such services may be provided infrequently.^37^ A 2010 Cochrane review identified 4 short-term, randomized controlled trials of postpartum family planning counseling interventions, 2 of which had insufficient sample size and/or data. The remaining 2 studies found that counseling leads to increased use of contraceptive methods.^36^ The trials took place in developed countries (Australia and the United States), all of the interventions were provided primarily by trained health professionals, and most counseling sessions were provided in a clinical setting, frequently in the hospital after delivery. In a non-randomized controlled trial in Chile, an integrated model of family planning promotion and infant care showed gains in infant care indicators and high rates of contraceptive acceptance in the intervention group, but the study was not designed to measure differences in contraceptive continuation between the intervention and comparison groups.^38^

Results of the study described in this article are currently being analyzed and will be published in a future paper.

### Number and Timing of CHW Visits

The HFS intends to fill a gap in the literature on postpartum family planning by providing guidance about a scaled-up package of integrated community-based care, including about the appropriate number and timing of CHW household visits. Our postpartum family planning intervention package included 1 antenatal visit and 4 postpartum visits during the first 5 months postpartum, plus 1 visit every 2 months for pregnancy surveillance and contraceptive distribution.

One antenatal and 3 postpartum visits are feasible and sustainable in many community-based MNH programs, including in Bangladesh. For example, the Maternal and Child Health Integrated Program (MCHIP) in Bangladesh funded by the U.S. Agency for International Development (known as the MaMoni project) is implementing an MNH program in 2 districts that involves 3 postpartum visits with integrated family planning counseling and services. Although the 5 CHW visits in the HFS intervention package may be considered intensive, preliminary results indicate that the large majority of women received the full number of scheduled visits and that the schedule did not compromise worker performance. Based on programmatic experience, the HFS team concluded that fewer visits would lead to gaps in continuation of breastfeeding and the transition from LAM to other modern contraceptive methods.

Although making 5 CHW visits may be considered intensive, preliminary results indicate it was feasible.

### Community-Based Distribution of Contraceptives

Doorstep delivery of contraceptives was not originally part of the HFS intervention package. The government of Bangladesh had moved away from community-based distribution of contraceptives toward clinic-based provision of family planning services.^39^-^41^However, HFS-enrolled women began requesting contraceptives from CHWs, and so we added contraceptive distribution to the study design as an opportunity to potentially enhance intervention impact. In this culturally conservative area, women's movement outside the home is curtailed, even more so than in other areas of Bangladesh, which limits their contraceptive access.

### Building on Lessons Learned From Other Community-Based Integrated Programs

The HFS builds on important lessons from the Bangladesh Matlab Family Planning–Health Services Project^42^ and the Navrongo research in Ghana^43^—both of which provided integrated family planning and maternal and child health services—but focuses on previously neglected areas and new emerging public health issues.

#### Type of Health Care Provider

The HFS relies on service delivery through locally recruited CHWs with a grade 10 education. In contrast, the Navrongo project achieved results by using more highly educated health care professionals—nurses—a cadre not widely available in many developing countries.

#### Targeting of Services to Pregnant and Postpartum Women

The HFS targets family planning services to pregnant and postpartum women—an underserved group of women with demonstrated high unmet need for family planning—not to all women in the community, as with the Matlab^42^ and Navrongo projects. It also reaches first-time mothers, who are at higher risk than women with 2 or 3 births. In 2005, a literature review identified 27 integrated programs at the community level, and only 5 focused on the postpartum period.^44^ Focusing efforts on postpartum women also might improve consistent CHW household visits; Matlab field workers were supposed to visit all eligible women every 3 months, but they averaged only 1.5 visits per year per woman.^39^

#### Improving Family Planning Continuation Rates

The Matlab project demonstrated progress in improving contraceptive continuation rates,^5^ but first-year discontinuation rates are still high in Bangladesh. At the time of the HFS design, 1-year contraceptive discontinuation rates had increased from 49% in 2004 to 57% in 2007.^3^ The HFS evaluation is examining the extent to which the intervention motivates women to continue using family planning for at least 2 years without interruption.

#### Lengthening Birth-to-Pregnancy Intervals

Matlab made progress in helping women to space their pregnancies, yet currently in Sylhet, 47% of second-order and higher births are spaced less than 36 months apart, and for the 15–19 age group nationally, this percentage rises to 80%.^6^ HFS communication messages focus specifically on preventing high-risk pregnancies by increasing birth-to-pregnancy intervals, in part through use of LAM—a method for which efficacy studies were carried out only relatively recently, between 1997 and 2000. Thus, LAM was unavailable when Matlab and Navrongo were carrried out.

#### Newborn Mortality

Navrongo demonstrated reductions in child mortality,^43^ but by the 1990s under-5 mortality in most developing countries was increasingly dominated by newborn mortality, which called for a different set of interventions. HFS includes interventions to reduce newborn and infant mortality by focusing on the 1-year extended postpartum period.

#### Sequencing of Integration Activities

The HFS design adds postpartum family planning education and services into an ongoing and successful maternal and newborn care program, whereas with the Matlab project, maternal and child health services were gradually added to the responsibilities of the family planning field worker.

The findings from the HFS trial are anticipated to help practitioners provide women and infants in low-resource settings with an integrated, community-based continuum of care.[Fig f02][Fig f03][Fig f04]
